# The effect of topical decorin on temporal changes to corneal immune cells after epithelial abrasion

**DOI:** 10.1186/s12974-022-02444-8

**Published:** 2022-04-12

**Authors:** Mengliang Wu, Laura E. Downie, Lisa J. Hill, Holly R. Chinnery

**Affiliations:** 1 Department of Optometry and Vision Sciences, The University of Melbourne, Parkville, VIC Australia; 2 School of Biomedical Sciences, Institute of Clinical Sciences, University of Birmingham, Birmingham, B15 2TT UK

**Keywords:** Decorin, Small leucine-rich proteoglycan, Dendritic cells, Macrophages, Neutrophils, Nerve regeneration, Cornea, Wound healing

## Abstract

**Background:**

Corneal immune cells interact with corneal sensory nerves during both homeostasis and inflammation. This study sought to evaluate temporal changes to corneal immune cell density in a mouse model of epithelial abrasion and nerve injury, and to investigate the immunomodulatory effects of topical decorin, which we have shown previously to promote corneal nerve regeneration.

**Methods:**

Bilateral corneal epithelial abrasions (2 mm) were performed on C57BL/6J mice. Topical decorin or saline eye drops were applied three times daily for 12 h, 24 h, 3 days or 5 days. Optical coherence tomography imaging was performed to measure the abrasion area. The densities of corneal sensory nerves (β-tubulin III) and immune cells, including dendritic cells (DCs; CD11c^+^), macrophages (Iba-1^+^) and neutrophils (NIMP-R14^+^) were measured. Cx3cr1^gfp/gfp^ mice that spontaneously lack resident corneal intraepithelial DCs were used to investigate the specific contribution of epithelial DCs. Neuropeptide and cytokine gene expression was evaluated using qRT-PCR at 12 h post-injury.

**Results:**

In decorin-treated corneas, higher intraepithelial DC densities and lower neutrophil densities were observed at 24 h after injury, compared to saline controls. At 12 h post-injury, topical decorin application was associated with greater re-epithelialisation. At 5 days post-injury, corneal stromal macrophage density in the decorin-treated and contralateral eyes was lower, and nerve density was higher, compared to eyes treated with saline only. Lower expression of transforming growth factor beta (TGF-β) and higher expression of CSPG4 mRNA was detected in corneas treated with topical decorin. There was no difference in corneal neutrophil density in Cx3cr1^gfp/gfp^ mice treated with or without decorin at 12 h.

**Conclusions:**

Topical decorin regulates immune cell dynamics after corneal injury, by inhibiting neutrophils and recruiting intraepithelial DCs during the acute phase (< 24 h), and inhibiting macrophage density at the study endpoint (5 days). These immunomodulatory effects were associated with faster re-epithelialisation and likely contribute to promoting sensory nerve regeneration. The findings suggest a potential interaction between DCs and neutrophils with topical decorin treatment, as the decorin-induced neutrophil inhibition was absent in Cx3cr1^gfp/gfp^ mice that lack corneal epithelial DCs. TGF-β and CSPG4 proteoglycan likely regulate decorin-mediated innate immune cell responses and nerve regeneration after injury.

**Supplementary Information:**

The online version contains supplementary material available at 10.1186/s12974-022-02444-8.

## Background

Distinct populations of resident immune cells are distributed throughout the healthy mouse cornea, including intraepithelial dendritic cells (DCs), stromal macrophages and γδ T cells, the latter residing mostly in the peripherally located limbal epithelium [1–4]. With the development of transgenic animals and advances in imaging techniques, the role(s) of immune cells are becoming increasingly recognised in the contexts of corneal development [5], epithelial wound healing, corneal neuropathy and infectious keratitis [6, 7]. Recently, the phenotype and function of immune cells have been investigated in corneal neuropathy secondary to ocular and systemic diseases, consistent with a growing recognition of the importance of neuroimmune interactions in the cornea [8, 9].

The crosstalk between immune cells and the peripheral nervous system has been investigated in many tissues, including the gut, skin and lung [10, 11], and more recently in the cornea [12–14]. Peripheral sensory nerves play an important role in tissue homeostasis and wound healing, by responding rapidly to nociceptive stimuli and irritants, and modulating local immune responses. Reciprocally, immune cells can affect neuronal function by releasing cytokines and neurotransmitters, including acetylcholine [15, 16]. In the cornea, the sensory nerves originate from the ophthalmic division of the trigeminal ganglion. After penetrating the corneoscleral limbus to form the stromal nerve trunks, some nerve branches course from the anterior stroma and run parallel between the corneal basal epithelial cells and the anterior limiting lamina [17], forming the sub-basal nerve plexus (SBNP). Branches derived from the corneal SBNP penetrate vertically through the epithelium, terminating beneath the epithelial surface as superficial nerve terminals (SNT).

Decorin is a small leucine-rich proteoglycan that exists in many connective tissues. In the cornea, it contributes to collagen fibrillogenesis as well as collagen fibril spacing, which is critical for corneal transparency [18, 19]. Decorin consists of a core protein that contains a domain of tandem leucine-rich repeats, and a single glycosaminoglycan chain attached to the N terminus. Though the glycosaminoglycan chain is involved in some decorin–ligand interactions, most ligand-binding regions of decorin are located at its core protein [20]. It is reversible between homodimers and monomers of decorin, and the dimerisation is not necessary for decorin stabilisation [21]. Decorin has been illustrated to promote nerve axon growth following spinal cord injury in vivo [22, 23] and regulate inflammatory responses in in vitro studies [24, 25]. Our laboratory has previously demonstrated that decorin enhances corneal sensory nerve regeneration and inhibits macrophage recruitment one week after sterile epithelial injury, and was associated with a higher density of corneal intraepithelial DCs within 6 h after topical application [26].

Many diseases that involve corneal neuropathy show concurrent changes to corneal sensory nerves and local immune cells. A recent study revealed the functional significance of physical connections between intraepithelial DCs and corneal nerves in the pathogenesis of corneal herpetic infection [27] and following sterile abrasion injury [28]. Other studies have reported that in diabetic mice with corneal injury, improved nerve regeneration was associated with the recruitment of anti-inflammatory macrophages [29]. Further supporting a role for macrophages in corneal nerve recovery, Liu et al. reported that adoptive transfer of anti-inflammatory macrophages promoted corneal nerve regeneration after injury, in a mouse model of antibiotic-induced gut microbiota dysbiosis [30].

While our understanding of immune responses after corneal injury and their relationship to sensory nerve degeneration and regeneration has increased in recent years, key aspects remain poorly understood. Many current studies relating to corneal neuroimmune interactions are based on a specific study timepoint, with temporal changes rarely explored. The aims of this study were to investigate temporal alterations to corneal immune cells during wound healing after corneal injury, and to examine the effect that topical decorin application, which promotes nerve regeneration, has on this immune response. We also sought to determine which potential corneal cytokines and/or neuropeptides are modulated during these responses, in order to define the mechanisms that underpin these neuroimmune interactions within the injured cornea.

## Materials and methods

### Animals and corneal abrasion model

All animals were treated in accordance with the ARVO Statement for the Use of Animals in Ophthalmic and Vision Research, and all procedures were approved by the Animal Ethics Committee at the Florey Institute of Neuroscience and Mental Health (18-094-UM). Female C57BL/6J mice (6–8 weeks old) were obtained from the Animal Resources Centre, Murdoch, Western Australia and housed in a specific pathogen-free environment at the Florey Institute of Neuroscience and Mental Health. Cx3cr1-deficient (Cx3cr1^gfp/gfp^) mice that spontaneously lack resident corneal epithelial DCs [31] were also included in the study. Mice were anesthetised with an intraperitoneal injection of ketamine (80 mg/kg) and xylazine (10 mg/kg). A corneal abrasion injury was performed on both eyes of each animal as described previously [32]. Briefly, a 2-mm diameter circular area of the central corneal epithelium was demarcated using a sterile 2-mm trephine, then debrided using an ophthalmic burr (0.5 mm, Algerbrush II; Alger Equipment Co., Lago Vista, TX, USA). Immediately following the injury, a 2-µl drop of sterile saline was applied to each eye to prevent corneal drying.

### Spectral domain optical coherence tomography (SD-OCT)

In vivo SD-OCT imaging was performed to measure the corneal wound sizes immediately following the injury. Anesthetised mice were placed on the animal imaging mount and rodent alignment stage (AIM-RAS) attached to the SD-OCT imaging device (Bioptigen Envisu R2200 VHR; Bioptigen, Inc., Durham, NC, USA). Volumetric 3 × 3 mm rectangular scans of the central cornea (1000 A-scans/200 B-scans) were captured using an 18-mm telecentric lens immediately after the abrasion. En face images were used to measure the size of the epithelial abrasion area using a freehand trace tool in ImageJ software (http://imagej.nih.gov/ij/; provided in the public domain by the National Institutes of Health, Bethesda, MD, USA).

### Decorin dosage and topical treatment

The decorin used in this study is a recombinant human decorin core protein (Galacorin™, Catalent, USA). Our previous work has demonstrated a neuroregenerative effect with topical decorin dosed at a concentration of 4.76 mg/ml [26]. To consider whether a lower concentration might be effective, 4 µl of decorin in a range of concentrations (0.24 mg/ml, 1.07 mg/ml, and 4.76 mg/ml) was applied on one eye of treatment groups three times per day for 1 week after the corneal abrasion (*n* = 8 for each concentration), with the contralateral eyes treated with saline (results shown in Additional file 1: Fig. S1).

The decorin concentration of 1.07 mg/ml was chosen for the subsequent experiments, as improved corneal nerve regeneration in both SBNP and SNT was observed after topical decorin treatment with this concentration. Mice received eye drops (one randomly selected eye treated with decorin and the other eye received saline, *n* = 8, or both eyes treated with saline for the control group, *n* = 8) either three times administered at four hourly intervals over a 12-h period or three times per day for 1, 3, or 5 days. To investigate corneal RNA expression, a separate treatment group (*n* = 16) and a control group (*n* = 8) with the same eye drop protocol for the 12-h timepoint were included. En face images from SD-OCT scans were obtained at time 0 and 12 h to assess the injured epithelium area at 0 h, and to quantify percentage of re-epithelialisation at the endpoint. All mice were gently held for one minute after each eye drop, to allow the eye drops to distribute across the ocular surface before the mice were returned their cages.

### Cx3cr1^gfp/gfp^ mice model

Cx3cr1^gfp/gfp^ mice spontaneously lack resident corneal epithelial DCs [31]. To investigate the potential role of resident and early infiltrating DCs on corneal neutrophil recruitment, Cx3cr1^gfp/gfp^ mice were examined 24 h after a 2-mm corneal epithelial injury and topical application of either decorin or saline eye drops, administered three times daily (one randomly selected eye treated with decorin and the other eye received saline, *n* = 5, or both eyes treated with saline for the control group, *n* = 5).

### Corneal wholemount immunofluorescence

Mice were euthanised at the designated experimental timepoints and enucleated eyes were fixed in chilled acetone for 20 min and then washed in phosphate buffered saline (PBS). Dissected corneas were incubated in 20 mM ethylenediaminetetraacetic acid for 30 min at 37 °C and then blocked with 3% bovine serum and 0.3% Triton X-100 in PBS for 1 h at room temperature. For immunostaining, tissues were incubated overnight at 4 °C with primary antibody rabbit anti-β tubulin 488 (1:500; #AB15708A4, Millipore, Billerica, MA, USA), hamster anti-CD11c (1:50; N418, Abcam, Cambridge, MA, USA), rabbit anti-IBA1 (1:500; #019-19741, Fujifilm Wako Chemicals, Osaka, Japan), rat anti-CD45 (1:500; #550539, BD Biosciences, Franklin Lakes, NJ, USA), or rat anti-NIMP (1:100; NIMP-R14, Abcam, Cambridge, MA, USA). Afterwards, tissue flat mounts were washed with PBS three times before incubation with the secondary antibodies, goat anti-rabbit Alexa Fluor 647 (1:500; #A21244, ThermoFisher Scientific, Carlsbad, CA, USA), goat anti-rat Alexa Cy3 (1:500; #A10522, ThermoFisher Scientific, Carlsbad, CA, USA), goat anti-hamster 488 (1:500; #ab173003, Abcam, Cambridge, MA, USA) and Hoechst (1:1000; Sigma, St Louis, MO, USA) for 2 h at room temperature. Immunostained samples were then washed and mounted onto glass slides with aqueous mounting medium and coverslipped for imaging.

### Corneal nerve and immune cell image acquisition and analysis

Corneal wholemounts were imaged using a confocal microscope (Confocal Laser Scanning Microscopy SP8; Leica Microsystems, Buffalo Grove, IL, USA). Three non-overlapping z-series were captured from the central (within the 0.75 mm radial area from the corneal apex) and peripheral cornea (between 1 and 1.5 mm radial area from the corneal apex), respectively. For nerve quantification, separate *z*-stacks of the SNT and SBNP were created by generating *z*-projections of the superficial and basal epithelial layers [32, 33]. To compare the immune cell changes between groups, *z*-stack images of the anterior corneal stroma (5 µm directly below the basal epithelium) were created for analysing neutrophils (NIMP^+^ with a distinct polymorphonuclear appearance) and macrophages (CD45^+^ Iba1^+^). For the analysis of DCs (CD45^+^ CD11c^+^), epifluorescent images (two central and two peripheral) were collected using an Olympus BX51 microscope (450 µm × 300 µm area). All images were analysed by a masked observer. The percentage area occupied by nerves was evaluated using manual thresholding in ImageJ software [32]. The density of corneal macrophages, neutrophils and DCs was counted manually using ImageJ.

### RNA extraction and quantitative real-time RT-PCR

Two corneas that received the same intervention were pooled together to obtain a sufficient amount of RNA for analysis. Tissues were homogenised in lysis buffer with a 5-mm bead using TissueLyser II® (QIAGEN, MD, USA). Total RNA was extracted using PureLink® RNA Mini Kit according to the manufacturer’s instructions. Complementary DNA (cDNA) was generated using Tetro cDNA Synthesis Kit (Bioline, London, UK). A ‘No reverse transcriptase’ control was included.

Gene expression alterations were detected using duplex quantitative real-time PCR assays (with FAM or VIC fluorescent reporter dyes) for β-actin (Mm01205647_g1), IL-1β (Mm00434228_m1), TNF-α (Mm00443258_m1), TGF-β (Mm00441724_m1), NGF (Mm00443039_m1), CNTF (Mm00446373_m1), CGRP (Mm00801463_g1), TLR2 (Mm00442346_m1), CXCL2 (Mm00436450_m1) and CSPG4 (Mm00507257_m1) (Applied Biosystems, ThermoFisher Scientific, Carlsbad, CA, USA) and Taqman Gene Expression Master Mix (Applied Biosystems). Quantitative real-time RT-PCR assay was conducted with Vii7A system (Applied Biosystems). The relative quantity of mRNA expression was calculated using the 2^−ΔΔCt^ formula, normalised to the housekeeping gene.

### Statistical analyses

For the mice who received different treatments in each eye, the data analysis was performed by fitting a mixed-effects model taking account the intraclass correlation for using data from both eyes. After fitting the model, post hoc tests were performed to examine the direct effects of topical decorin and any potential ‘contralateral eye effect’ from the decorin intervention. For analysis of qPCR results, one-way ANOVA followed by a Tukey’s post hoc test was performed. All statistical analyses were performed in Stata software (version 14.2; StataCorp LLC, College Station, TX, USA). A *p* < 0.05 was considered statistically significant. All summary data are shown as mean ± standard error of the mean (SEM).

## Results

### Corneal abrasion injury

The initial corneal abrasion injury was measured for each animal to ensure similar wound sizes were generated in all eyes. There was no inter-group or inter-batch differences in the injury size before the topical treatments were administered (see Additional file 1: Fig. S2).

### Corneal sensory nerve regeneration

**Fig. 1:**
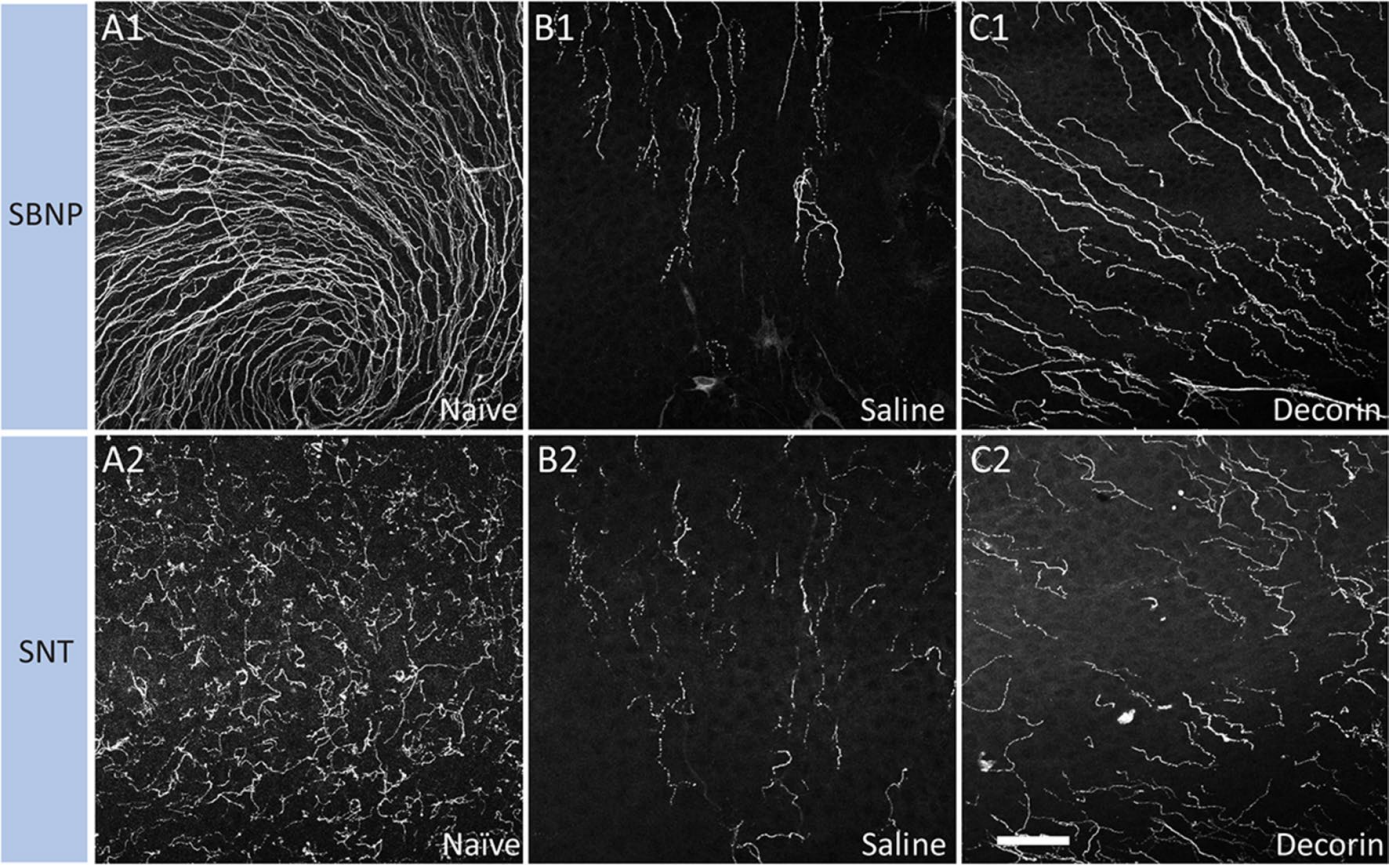
Representative confocal microscopic images of the SBNP and overlying SNT in the central region in an uninjured cornea (**A1**, **A2**) or injured cornea treated with topical saline (**B1**, **B2**) or decorin (**C1**, **C2**) at 5 days after corneal abrasion. Scale bar in **C2** (50 µm) applies to all images

**Fig. 2:**
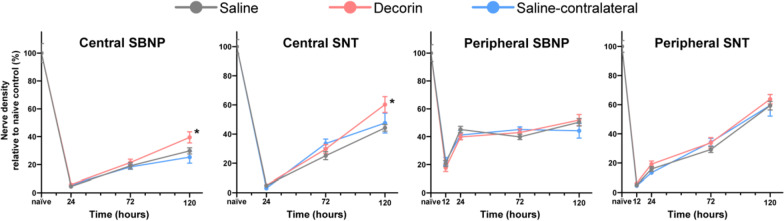
Corneal nerve regeneration after epithelial abrasion injury, during a 5-day treatment period with topical decorin or saline, dosed three times/day. Naïve refers to intact, untreated corneas. *Indicates a statistically significant difference between eyes with and without decorin treatment. *SBNP* sub-basal nerve plexus; *SNT* superficial nerve terminals. *n* = 8 mice per group, data represent mean ± SEM

Corneal sensory nerve density in the central cornea was reduced dramatically after the epithelial injury. The damaged nerve plexi regenerated gradually, with approximately 40% of the SNT and 30% of the SBNP nerve regenerated after 5 days (Figs. 1, 2). Overall, eyes treated with topical decorin showed greater corneal nerve regeneration in the central cornea compared to those treated with saline at 5 days (SBNP, *p* = 0.042; SNT, *p* = 0.014). In the peripheral cornea (outside of the wound zone), relative to baseline only 5% of SNT but 20% of SBNP was  present at 12 h after the injury. The peripheral SNT regenerated rapidly afterwards and reached 60% of pre-injury levels after 5 days, while the peripheral SBNP recovered to 40% by 1 day post-injury and then the nerve regeneration slowed. In the peripheral corneal SNT and SBNP, there was no significant difference between decorin- and saline-treated eyes at any of the evaluated timepoints.

### Corneal immune cell densities in the epithelial abrasion model

**Fig. 3:**
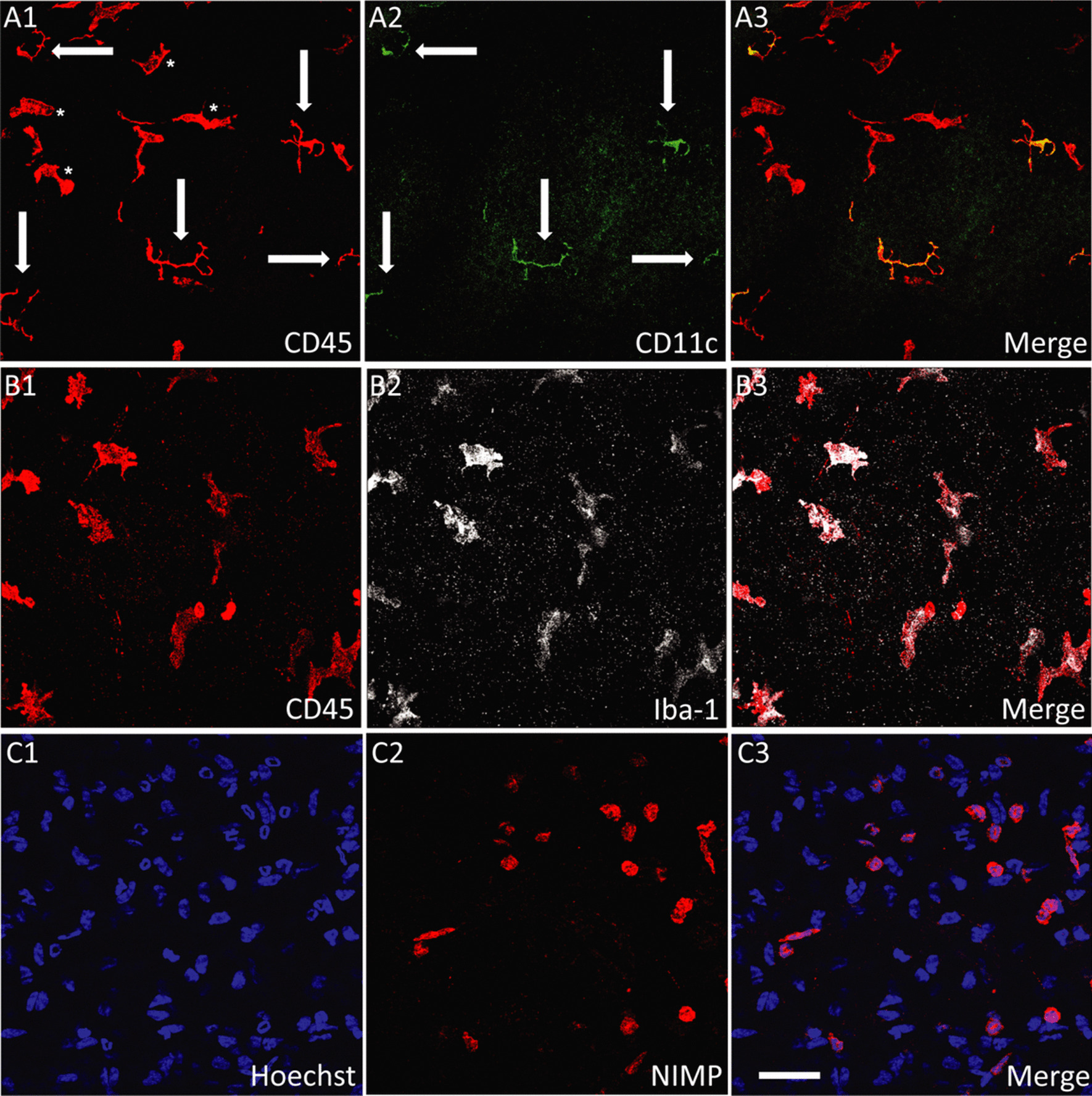
Representative confocal microscopic images of the intraepithelial CD45^+^ CD11c^+^ DCs (**A1**–**A3**), anterior stromal CD45^+^ Iba1^+^ macrophages (**B1**–**B3**) and infiltrating NIMP^+^ neutrophils (**C1**–**C3**) in the injured cornea. Arrows indicate intraepithelial CD45^+^ CD11c^+^ DCs in the epithelium, asterisks indicate CD45^+^ CD11c^−ve^ cells located in the anterior stroma. Scale bar in **C3** (50 µm) applies to all images

**Fig. 4:**
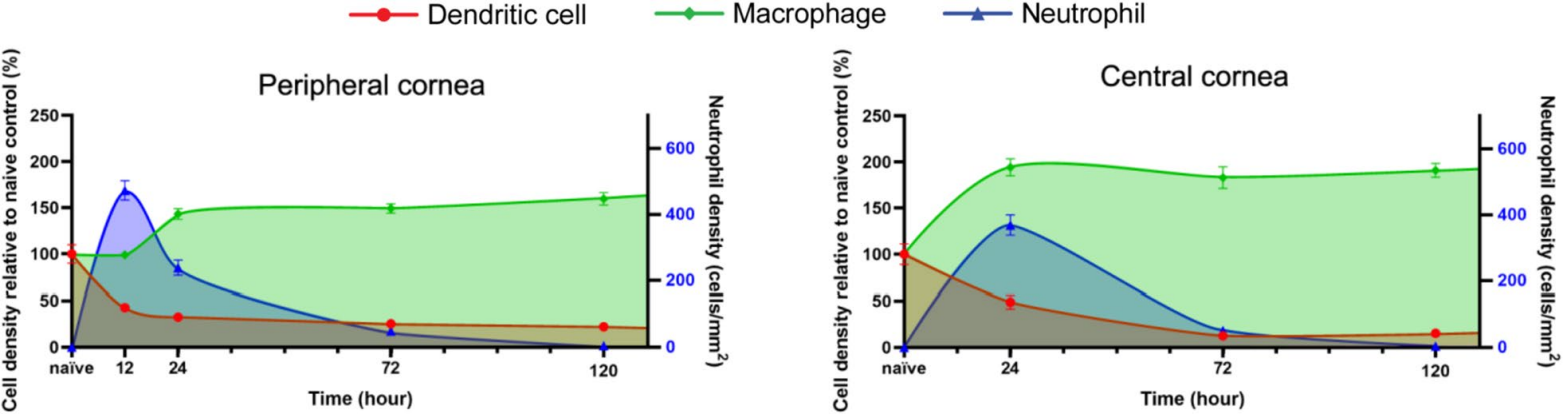
Temporal changes to epithelial dendritic cells, stromal macrophages and stromal neutrophil densities following a central 2-mm corneal epithelial abrasion injury. *n* = 8 mice per group, data represent mean ± SEM

Corneal immune cells were quantified in the central and peripheral cornea at each time point (Fig. 3), except at 12 h when the abraded epithelium had not fully regenerated. In the peripheral cornea, DC density was lower after the abrasion injury compared to naïve corneas, and remained low during the following 5 days (Fig. 4A). Infiltrating neutrophil numbers peaked at 12 h and were substantially lower by day 3, with no neutrophils present at day 5. Peripheral corneal macrophage density was similar in intact and injured corneas at 12 h, but was higher 1 day after the injury and the density remained elevated through to day 5. Similar changes to the immune cell dynamics were also observed in the central cornea (Fig. 4B).

### Effect of topical decorin on the temporal dynamics of corneal immune cells

**Fig. 5:**
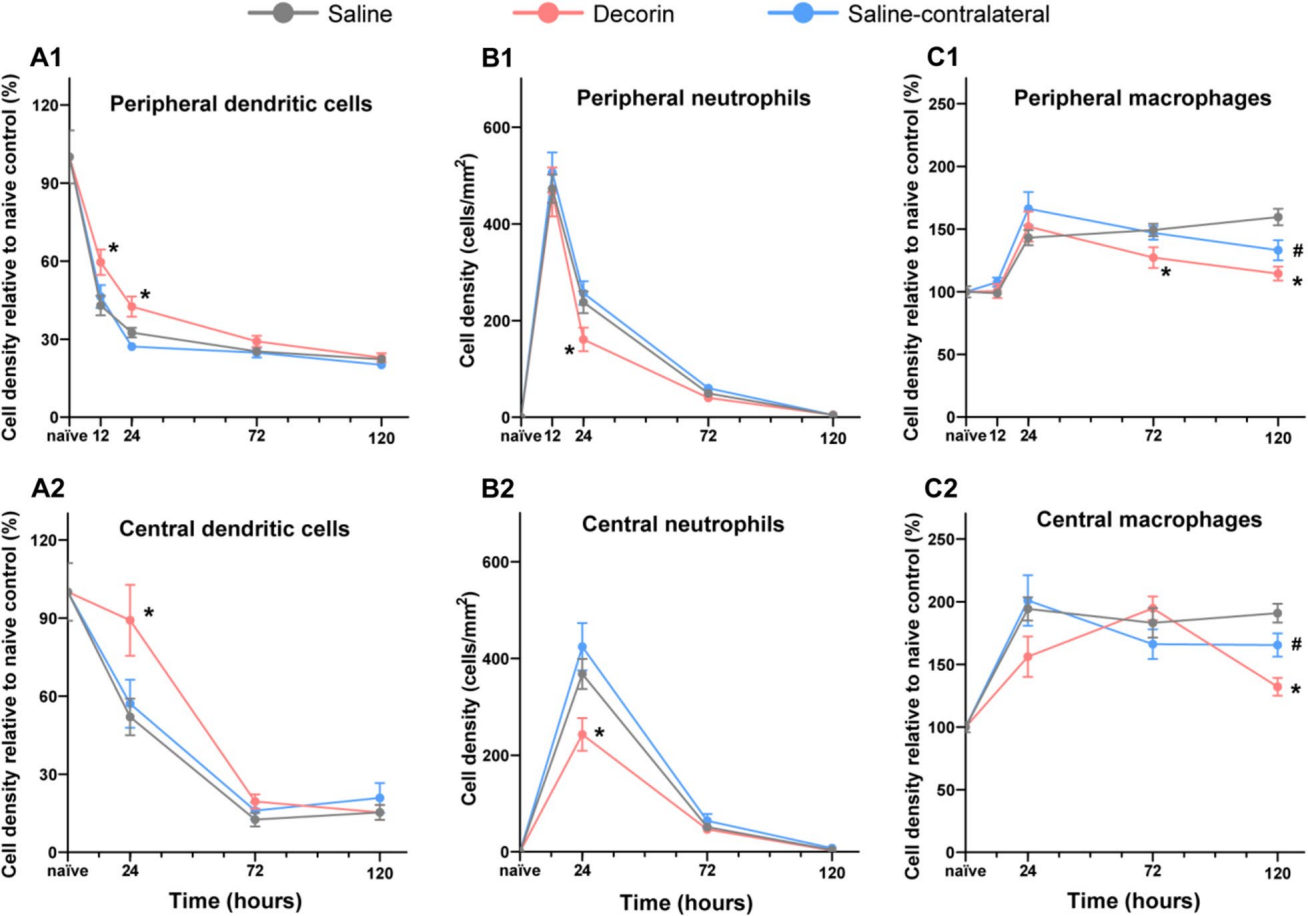
Temporal dynamics of corneal immune cells during 5 days of treatment with topical decorin after the central corneal abrasion. **A1**, **A2** The density of intraepithelial DCs was higher in decorin-treated eyes at 24 h compared to saline controls. **B1**, **B2** There was a lower density of neutrophils in decorin-treated eyes at 24 h after it peaked at 12 h in the peripheral cornea. **C1**, **C2** The density of corneal macrophages was lower in decorin-treated eyes and their contralateral eyes at 5 days after corneal injury. *Indicates a statistically significant difference between eyes treated with decorin and control eyes with saline. ^#^Indicates a statistically significant difference between the contralateral eyes from decorin-treated animals and saline control eyes

In decorin-treated eyes after the abrasion, a higher density of corneal DCs was observed at 12 h and 24 h compared with the saline (control) group (*p* = 0.029 and 0.009, respectively), while an inter-group difference was not observed at day 3 or 5 (Fig. 5A1, A2). The neutrophils infiltrated rapidly into the peripheral cornea at 12 h but there was no difference after treatment with decorin (Fig. 5B1, B2). However, decorin-treated eyes demonstrated a faster clearance of neutrophils, leading to a significantly lower density of neutrophils in the central and peripheral cornea at 24 h (*p* = 0.026 and *p* = 0.046, respectively). By day 3, there was no difference in neutrophil numbers between the groups.

With respect to macrophages, both central and peripheral corneal macrophage density was similar, at the acute stage, between decorin- and saline-treated eyes (Fig. 5C1, C2). The density of macrophages was significantly lower in decorin-treated eyes at day 3 (peripheral, *p* = 0.017) and day 5 (central and peripheral, *p* < 0.001 and *p* = 0.001, respectively). A lower number of macrophages was also observed in the contralateral eyes of decorin-treated eyes compared to saline-treated (control) eyes (central and peripheral, *p* = 0.041 and *p* = 0.026, respectively), suggesting a contralateral eye effect of decorin on macrophage activity.

### Effect of decorin on re-epithelialisation after corneal abrasion

**Fig. 6:**
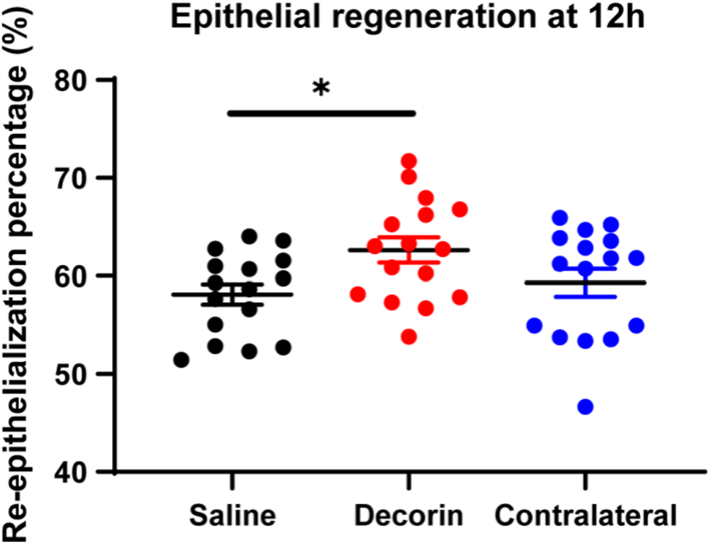
Corneal epithelial regeneration at 12 h after central corneal abrasion. *Indicates a statistically significant difference between eyes treated with decorin and control saline. Each data point represents one cornea, data represent mean ± SEM

At time 0 h (i.e., immediately after induction of the injury), the epithelial abrasion area was similar between decorin-treated eyes, contralateral eyes and saline-treated eyes (3.58 ± 0.36, 3.58 ± 0.24 and 3.59 ± 0.29 mm^2^, respectively). After three administrations of topical treatment (i.e., decorin or saline eye drops) over 12 h, a higher percentage of re-epithelialisation was observed in the decorin-treated eyes compared to control (saline-treated) eyes (Fig. 6, *p* = 0.038). There was no contralateral eye effect of decorin on the extent of corneal re-epithelialisation.

### Effect of topical decorin on corneal neutrophil infiltration in Cx3cr1^gfp/gfp^ mice

To determine if the lower neutrophil responses in decorin-treated eyes were related to the higher density of corneal DCs observed at 12 and 24 h post-injury, Cx3cr1^gfp/gfp^ mice lacking both resident and infiltrating DCs in the mouse corneal epithelium were investigated. There was no significant difference in the density of central or peripheral neutrophils between decorin- and saline-treated eyes at 24 h, indicating that corneal epithelial DCs may regulate the decorin-enhanced neutrophil clearance (see Additional file 1: Fig. S3). Consistent with the wild-type mice, the density of corneal macrophages was similar in the eyes treated with decorin compared to those that received topical saline treatment.

### mRNA expression of neuropeptides and cytokines

**Fig. 7:**
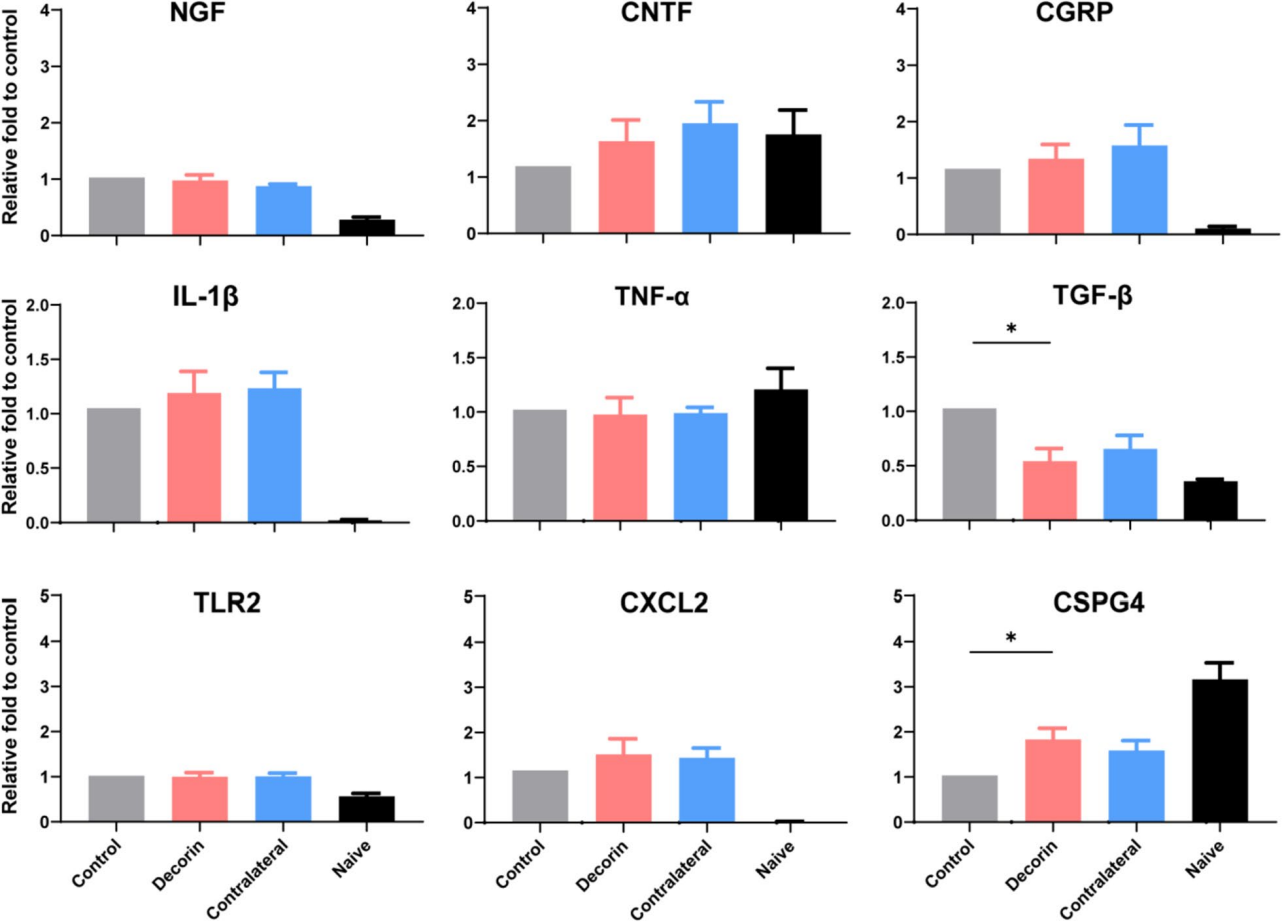
Corneal mRNA expression of neuropeptides and cytokines. *n* = 8 for each experimental group (control, decorin or contralateral group). Data from naive corneas (*n* = 3) are shown as a reference but are not included in the statistical analysis. *p < 0.05. *NGF* nerve growth factor; *CNTF* ciliary neurotrophic factor; *CGRP* calcitonin gene-related peptide; *TNF-α* tumour necrosis factor alpha; *TGF-β* transforming growth factor beta; *TLR2* toll-like receptor 2; *CXCL2* chemokine ligand 2; *CSPG4* chondroitin sulphate proteoglycan 4

Corneal inflammatory cytokine and neuropeptide gene expression were measured with quantitative real-time PCR using the 2^−ΔΔCt^ method. Among the detected cytokines and proteins, a downregulation in TGF-β and a upregulation of CSPG4 were observed with topical application of decorin, compared to control (saline-treated) eyes. There was no significant difference in the mRNA expression of NGF, CGRP, CNTF, IL-1β, TLR2, TNF-α or CXCL2 (Fig. 7).

## Discussion

As one of the major components of the ocular surface barrier, the cornea is susceptible to physical, chemical and microbial insults. The corneal immune system, in particular the innate immune cells, plays an important role in the defense against various threats by coordinating innate inflammatory responses [34, 35]. However, over-reactive and/or unresolved inflammatory responses can be harmful to local cells and tissues. For example, studies have proposed that ongoing inflammatory responses and elevated cytokines in the cornea after Herpes Simplex Virus-1 (HSV-1) infection contribute to corneal denervation [36, 37]. Moreover, a faster resolution of the inflammatory response may be associated with enhanced corneal nerve regeneration post-infection [38]. The present study is the first to demonstrate the temporal dynamics of corneal immune cell infiltration after epithelial injury, in eyes treated with either saline (vehicle) or topical decorin. Consistent with our previous findings [26], 5 days of topical treatment with decorin promoted sensory nerve regeneration in the central corneal after corneal abrasion injury. Extending these findings, the current investigations demonstrated more rapid epithelial wound healing in decorin-treated corneas, and alterations to the expression of TGF-β and CSPG4 mRNA after topical decorin treatment. Together, these findings provide strong evidence for crosstalk between corneal immune cells and sensory nerves during corneal wound healing.

Several mouse studies have demonstrated the presence of resident corneal intraepithelial DCs, which are more abundant in the peripheral cornea, and less populous in the central cornea [31, 39]. The density of corneal DCs increases in many ocular and systemic diseases involving chronic inflammation, including experimental dry eye disease [40] and diabetes [14, 41]. In our previous report on the effect of decorin on corneal nerve recovery, decorin treatment was associated with greater DC recruitment at six hours post-injury. To further evaluate the temporal dynamics of DCs in the epithelium in response to decorin, in the present study we assessed DC density at later time points and found a higher cell density in decorin-treated eyes relative to saline-treated controls during the acute-phase; this finding may be related to the faster re-epithelialisation observed at 12 h after injury. Gao et al. reported that the local depletion of corneal and conjunctival DCs delays epithelial wound closure, indicating an interplay between DCs and epithelial cells during corneal wound healing [42]. This is also supported by the difference in DC density only being observed during the first 24 h, a time at which re-epithelialisation is occurring.

There is emerging evidence for reciprocal communication between corneal DCs and nerves during both homeostasis and inflammatory conditions. In healthy human corneas, a higher DC density (greater than 50 cells/mm^2^) in the central cornea is strongly correlated with higher corneal nerve density [43]. In animal models of diabetes (Leppin et al. [41]) and human patients with diabetes, lower corneal nerve fibre density is associated with higher corneal DC density [8, 44]. Corneal DCs in patients with diabetes were more likely to be arranged in a cluster at the level of the SBNP [45], suggesting that the activation of DCs may be associated with corneal nerve fibre damage. However, in streptozotocin-induced diabetic mice recovering from a corneal epithelial debridement wound, a lower density of intraepithelial DCs was associated with delayed sensory nerve regeneration compared to animals without diabetes [46]. Thus, the precise contribution of corneal epithelial DCs in corneal wound healing remains unclear. Our recent study was the first to demonstrate a DC-dependent neuroregenerative effect with topical decorin, 7 days after injury, using Cx3cr1^gfp/gfp^ mice that lack resident and infiltrating corneal DCs [26]. In chronically inflamed human corneas, enlarged, “activated” DCs are frequently proposed to be a driver of sensory nerve pathology [47], with an inverse relationship between corneal DC density and nerve fibre density reported in dry eye disease and Herpes Zoster Ophthalmicus (HZO) [48, 49]. The precise links between decorin, dendritic cells and the improved corneal epithelial and nerve regeneration remains unknown. It is possible that the higher density of corneal intraepithelial DCs in the acute-phase of corneal wound healing may improve corneal re-epithelialisation, thereby providing greater anatomical structural support for subsequent nerve regeneration.

Resident macrophages are distributed throughout the corneal stroma [50, 51] and participate in innate immune responses by phagocytosing cellular debris and secreting inflammatory cytokines to assist with tissue remodelling [52]. Macrophages have both pro-inflammatory and anti-inflammatory phenotypes [53]. Pro-inflammatory macrophages are thought to play an important role during the early stages of corneal wound healing by enhancing the inflammatory response, while anti-inflammatory macrophages suppress immune responses during the later stages of healing [29, 51]. Although the corneal macrophage phenotypes were not assessed in this study, an increased density of macrophages was observed 24 h after the injury in both saline- and decorin-treated corneas. The topical application of decorin appeared to accelerate the resolution of inflammation, as evidenced by a lower macrophage density after corneal injury; interestingly, a contralateral eye effect was apparent, despite using a fourfold lower dose in the current study compared to our previous report [26]. Evidence exists for contralateral effects related to corneal inflammatory responses in primarily unilateral ocular conditions, including infectious keratitis, HZO, and unilateral corneal nerve cut [49, 54, 55]. Thus, if pro-inflammatory signals can involve contralateral eye responses, it is feasible that this may extend to anti-inflammatory signals too. Another possible explanation is that the improved sensory nerve regeneration restores the ocular surface microenvironment and thus reduce local inflammatory signals that recruit macrophages to both eyes.

Cross-talk between macrophages and corneal nerves during wound healing has also been reported in recent years. In the peripheral cornea, where sensory nerves enter the cornea as large multifibre trunks before branching and innervating the central epithelial layers, intimate physical connections exist between corneal macrophages and these peripheral stromal nerve trunks [13]. Anti-inflammatory macrophages play a vital role in corneal nerve regeneration after superficial injury [30, 56]. Interestingly, Chucair-Elliott et al. reported that corneal nerve structure and function were preserved after corneal HSV infection in mice when macrophages were inducibly depleted [57], suggesting the inhibition of pro-inflammatory macrophages might be beneficial for encouraging nerve regeneration. However, considering the contralateral eye effect of decorin in our study was only observed with respect to macrophage density, and did not extend to nerve regeneration, the macrophage–nerve interaction might be not the only pathway contributing to the promoted nerve regeneration.

Neutrophils are the dominant infiltrating immune cell in the acute phase of inflammatory responses to sterile corneal injury, extravasating from the limbal vessels within hours of epithelial injury and migrating through the stroma where they contribute to corneal re-epithelialisation [58, 59]. However, it is notable that the contribution of neutrophils to tissue repair is short-lived, with these cells undergoing apoptosis and eventually being cleared by macrophages [60]. Our results demonstrated that topical decorin led to lower neutrophil recruitment during the acute phase after corneal injury, which may partially account for the improved corneal re-epithelialisation at 12 h. The effect of topical decorin on neutrophil attenuation may also be associated with the reduced gene expression of TGF-β. Decorin can bind to TGF-β to reduce its bioavailability and compete with its receptors to inhibit signal transduction [61, 62]. Furthermore, the neutrophil is one of the main sources of TGF-β production [63] thus the lower neutrophil density may explain the lower mRNA level of TGF-β observed in the present study. To further investigate if the decorin-induced neutrophil inhibition was associated with corneal DCs, we applied topical decorin following corneal abrasion in Cx3cr1^gfp/gfp^ transgenic mice that spontaneously lack intraepithelial DCs. The application of decorin to the injured corneas of Cx3cr1^gfp/gfp^ mice did not lower the neutrophil density, indicating that the intraepithelial DCs may be important in regulating neutrophil recruitment to the inflamed cornea.

Unlike the significantly altered density of corneal immune cells after topical application of decorin, expression of IL-1β, TNF-α and CXCL2 mRNA was similar across all treatment groups at 12 h post-injury. A possible explanation is that those cytokines can be produced by both immune and non-immune cells [64, 65]. Furthermore, some cytokines are mainly expressed by activated macrophages [66], which were less affected by decorin during the acute-phase post-injury. Despite the enhanced corneal nerve regeneration after topical decorin treatment, similar levels of NGF, CNTF and CGRP mRNA were observed, suggesting that decorin exerts neuroregenerative effects by regulating other neuropeptides or via pathways not examined in this study. The qPCR results in the current study show higher CSPG4 expression in injured eyes treated with decorin, compared to the saline control group. CSPG4 is a chondroitin sulphate proteoglycan, also known as neuron-glial antigen 2 (NG2) in rats [67]. Both in vitro and in vivo studies have demonstrated that NG2 inhibits neurite growth in the central nervous system after injury [68, 69]. However, in the peripheral nervous system, NG2 is unlikely to be a major inhibitor or promotor of axon regeneration or functional recovery after injury [70]. After injury, we observed a lower expression of CSPG4 mRNA in all groups compared to naïve corneas; however, compared to saline, decorin-treated corneas had a relatively higher expression of CSPG4 that was accompanied by a faster re-epithelialisation rate. CSPG4 is expressed by non-myelinating Schwann cells that are present in the corneal stroma, just beneath the epithelial basement membrane [71]. As corneal epithelial cells may function as surrogate Schwann cells for sensory nerve fibres in the epithelium [72], it is possible that the decorin-mediated accelerated epithelial recovery at 12 h explains this slightly higher expression of CSPG4 in response to decorin. Further studies are required to explore the location and function of CSPG4 in the cornea during homeostasis and in response to injury, to determine if the elevated CSPG4 gene expression is related to improved corneal wound healing.

Many studies have reported using solid lipid nanoparticles or liposomes with a high biocompatibility to deliver therapeutic molecules to the eye [73, 74]. Future studies using innovative ocular nanosystems to deliver decorin could be explored, as a basis for considering how this therapy might be ultimately translated into clinical practice. Furthermore, although a control group treated with vehicle solution (saline) was included in this study, it is possible that decorin, as a proteoglycan, may provide additional lubricating benefits (e.g., a higher viscosity) to the ocular surface. Our previous study has compared the therapeutic effects of decorin to a high viscosity gellan-based fluid gel and demonstrated that the fluid gel on its own did not offer any therapeutic benefits to corneal nerve regeneration [26]. Nevertheless, a positive control with lubricating eye drops used in clinical practice such as hyaluronic acid [75], should be included to verify the therapeutic effect of decorin in future clinical studies.

## Conclusion

This study demonstrates a distinctive pattern of temporal changes to immune cells after corneal epithelial injury that are modulated by topical application of decorin, which also promotes corneal nerve regeneration. These data provide evidence for potential interactions between corneal sensory nerves and immune cells including DCs, macrophages and neutrophils during wound healing. The contralateral eye effect of decorin on macrophage activity, but not epithelial recovery, neutrophil or DC recruitment, indicates there might be different mechanisms by which decorin affects immune cell subsets during corneal wound healing. The present findings provide rationale for future studies to investigate the detailed pathways of corneal neuroimmune crosstalk following treatment with decorin, as well as to determine the therapeutic potential of decorin in ocular and systemic diseases with chronic inflammation and corneal neuropathy.

## Supplementary Information

**Additional file 1: Figure S1.** Corneal nerve regeneration after topical application of 0.24, 1.07 or 4.76 mg/ml decorin at 1 week after corneal injury. **Figure S2.** Initial central corneal abrasion area at time 0 h, as measured using en face images of the injured corneas acquired using spectral domain optical coherence tomography. **Figure S3.** Corneal immune cell changes 24 h after injury and topical decorin treatment in Cx3cr1^gfp/gfp^ mice that spontaneously lack intraepithelial DCs.

## Data Availability

The datasets used and/or analysed during the current study are available from the corresponding author on reasonable request.

## Abbreviations

DC: Dendritic cell
HSV: Herpes Simplex Virus
HZO: Herpes Zoster Ophthalmicus
RT-PCR: Reverse transcription polymerase chain reaction
SBNP: Sub-basal nerve plexus
SD-OCT: Spectral domain optical coherence tomography
SNT: Superficial nerve terminal
TGF-β: Transforming growth factor beta
