# Supplementary material for: The effect of topical decorin on temporal changes to corneal immune cells after epithelial abrasion
Source: J Neuroinflammation. 2022 Apr 12;19:90. doi: 10.1186/s12974-022-02444-8 (PMC9006562; doi:10.1186/s12974-022-02444-8)
Supplement: Supplementary file 1 — Additional file 1: Figure S1. Corneal nerve regeneration after topical application of 0.24, 1.07 or 4.76 mg/ml decorin at 1 week after corneal injury. Figure S2. Initial central corneal abrasion area at time 0 h, as measured using en face images of the injured corneas acquired using spectral domain optical coherence tomography. Figure S3. Corneal immune cell changes 24 h after injury and topical decorin treatment in Cx3cr1gfp/gfp mice that spontaneously lack intraepithelial DCs. [file 12974_2022_2444_MOESM1_ESM.docx]

**Additional files**

**
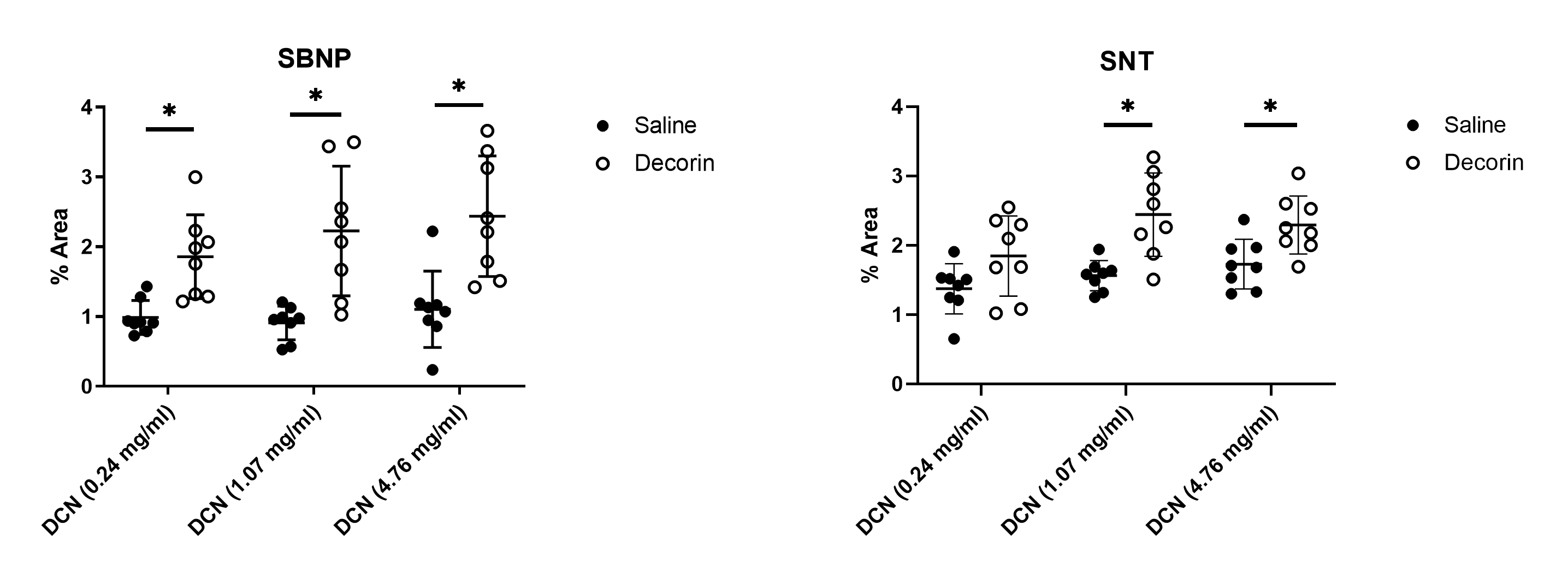
**

**Additional Figure S1:** Corneal nerve regeneration after topical application of 0.24, 1.07 or 4.76 mg/ml decorin at 1 week after corneal injury. Analysed by paired t-test in each group. SBNP, sub-basal nerve plexus; SNT, superficial nerve terminals; DCN, decorin. Each data point represents one eye. N=8 mice per group, data represent mean ± SD.

**
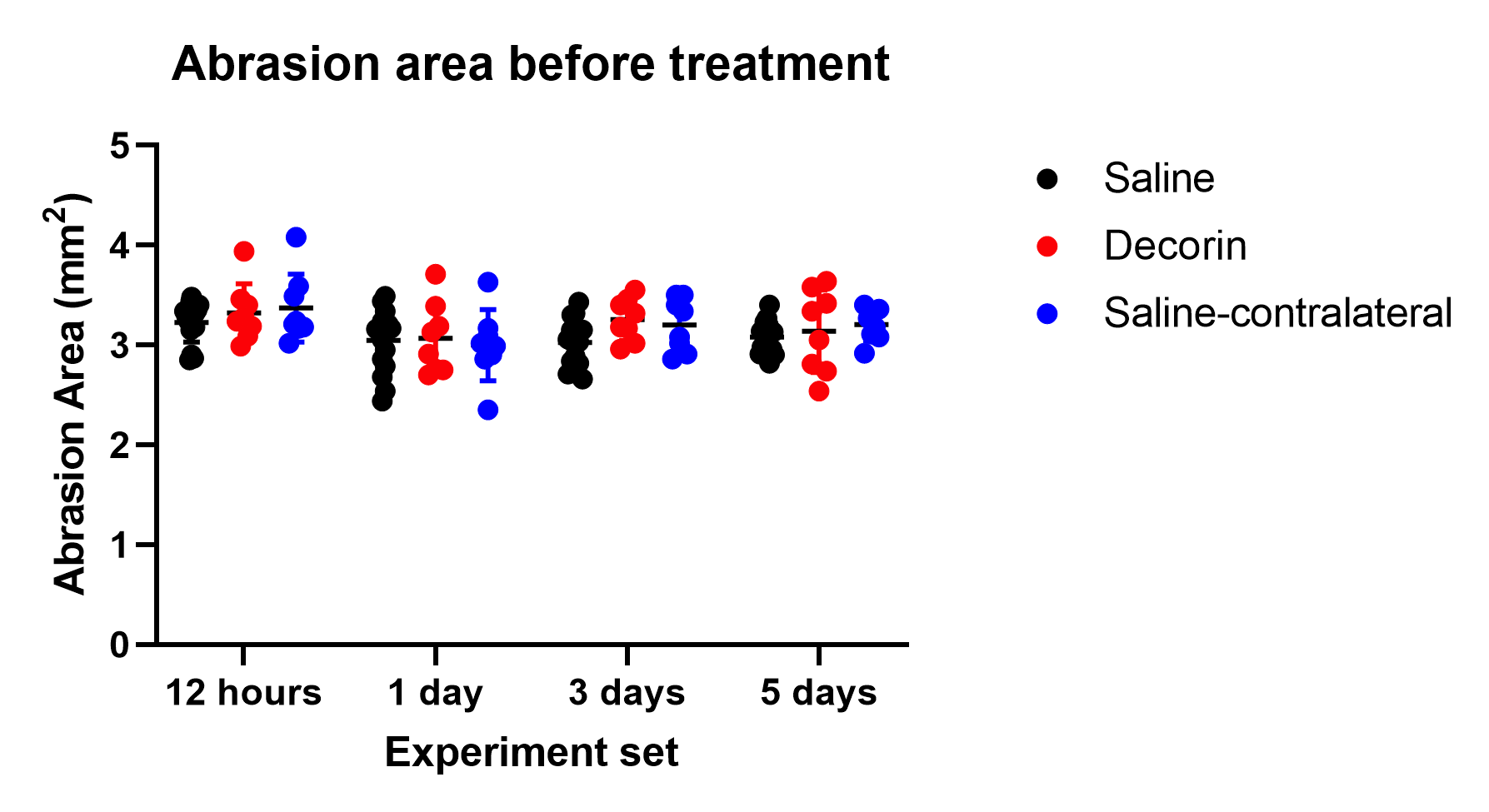
**

**Additional Figure S2:** Initial central corneal abrasion area at time 0 hours, as measured using *en face* images of the injured corneas acquired using spectral domain optical coherence tomography. Each data point represents one eye.

**
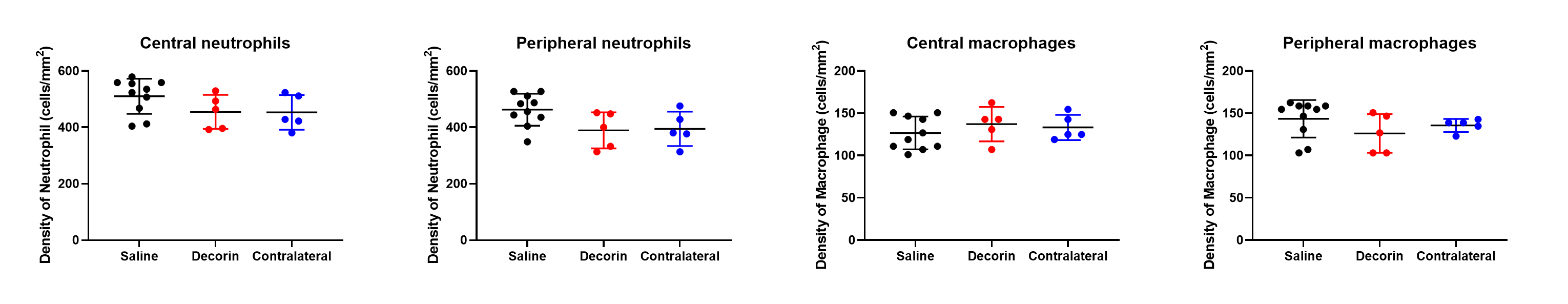
**

**Additional Figure S3:** Corneal immune cell changes 24 hours after injury and topical decorin treatment in Cx3cr1^gfp/gfp^ mice that spontaneously lack intraepithelial DCs. No difference was observed in the density of neutrophils or macrophages between the groups. Each data point represents one eye.
